# Bariatric surgery in individuals with severe cognitive impairment: report of two cases: Cirurgia bariátrica em indivíduos com déficits cognitivos graves: relato de dois casos

**DOI:** 10.1590/1516-3180.2016.0299071216

**Published:** 2017-04-20

**Authors:** Everton Cazzo, Martinho Antonio Gestic, Murillo Pimentel Utrini, Felipe David Mendonça Chaim, Elaine Cristina Cândido, Luciana Bueno da Silveira Jarolavsky, Ana Maria Neder de Almeida, José Carlos Pareja, Elinton Adami Chaim

**Affiliations:** I MD, MSc, PhD. Assistant Lecturer, Department of Surgery, Faculdade de Ciências Médicas da Universidade Estadual de Campinas (FCM-UNICAMP), Campinas (SP), Brazil.; II MD, MSc. Assistant Lecturer, Department of Surgery, Faculdade de Ciências Médicas da Universidade Estadual de Campinas (FCM-UNICAMP), Campinas (SP), Brazil.; III MD. Assistant Lecturer, Department of Surgery, Faculdade de Ciências Médicas da Universidade Estadual de Campinas (FCM-Unicamp), Campinas (SP), Brazil.; IV MD, MSc. Attending Physician, Department of Surgery, Faculdade de Ciências Médicas da Universidade Estadual de Campinas (FCM-UNICAMP), Campinas (SP), Brazil.; V BSc. Attending Nurse, Bariatric Surgery Outpatient Service, Hospital de Clínicas da Universidade Estadual de Campinas (HC-UNICAMP), Campinas (SP), Brazil.; VI BSc. Head Nurse, Bariatric Surgery Outpatient Service, Hospital de Clínicas da Universidade Estadual de Campinas (HC-UNICAMP), Campinas (SP), Brazil.; VII BSc. Attending Psychologist, Bariatric Surgery Outpatient Service, Hospital de Clínicas da Universidade Estadual de Campinas (HC-UNICAMP), Campinas (SP), Brazil.; VIII MD, PhD. Associate Professor, Department of Surgery, Faculdade de Ciências Médicas da Universidade Estadual de Campinas (FCM-UNICAMP), Campinas (SP), Brazil.; IX MD, MSc, PhD. Full Professor, Department of Surgery, Faculdade de Ciências Médicas da Universidade Estadual de Campinas (FCM-UNICAMP), Campinas (SP), Brazil.

**Keywords:** Prader-Willi syndrome, Down syndrome, Bariatric surgery, Obesity, Intellectual disability

## Abstract

**CONTEXT:**

Bariatric surgery has become the gold-standard treatment for refractory morbid obesity. Obesity is frequently associated with certain syndromes that include coexisting cognitive deficits. However, the outcomes from bariatric surgery in this group of individuals remain incompletely determined.

**CASE REPORT:**

A 25-year-old male with Prader-Willi syndrome, whose intelligence quotient (IQ) was 54, was admitted with a body mass index (BMI) of 55 kg/m^2^, associated with glucose intolerance. He underwent the Scopinaro procedure for biliopancreatic diversion, with uneventful postoperative evolution, and presented a 55% loss of excess weight one year after the surgery, with resolution of glucose intolerance, and without any manifestation of protein-calorie malnutrition. A 28-year-old male with Down syndrome, whose IQ was 68, was admitted with BMI of 41.5 kg/m^2^, associated with hypertension. He underwent Roux-en-Y gastric bypass, with uneventful postoperative evolution. He presented a 90% loss of excess weight one year after the surgery, with resolution of the hypertension.

**CONCLUSION:**

Bariatric surgery among individuals with intellectual impairment is a controversial topic. There is a tendency among these individuals to present significant weight loss and comorbidity control, but less than what is observed in the general obese population. The severity of the intellectual impairment may be taken into consideration in the decision-making process regarding the most appropriate surgical technique. Bariatric surgery is feasible and safe among these individuals, but further research is necessary to deepen these observations.

## RESUMO

**CONTEXTO:**

A cirurgia bariátrica tornou-se o tratamento padrão ouro para a obesidade mórbida refratária. A obesidade está frequentemente associada a certas síndromes nas quais também coexistem déficits cognitivos, entretanto, os resultados da cirurgia bariátrica nesse grupo de indivíduos ainda não foram completamente determinados.

**RELATO DE CASO:**

Um homem de 25 anos com síndrome de Prader-Willi, cujo quociente de inteligência (QI) era estimado em 54, foi admitido com índice de massa corporal (IMC) de 55 kg/m^2^, associado com intolerância à glicose. Foi submetido a uma derivação biliopancreática à Scopinaro, com evolução pós-operatória sem complicações significativas. Apresentou perda de 55% do excesso de peso um ano após a cirurgia, com resolução da intolerância à glicose, sem manifestação de desnutrição proteico-calórica. Outro paciente, homem de 28 anos com syndrome de Down, cujo QI era de 68, foi admitido com IMC de 41,5 kg/m^2^, associado a hipertensão arterial. Foi submetido ao *bypass* gástrico em Y de Roux, com evolução pós-operatória sem complicações. Apresentou perda de 90% do excesso de peso após um ano e resolução da hipertensão.

**CONCLUSÃO:**

A cirurgia bariátrica em indivíduos com déficits intelectuais é um tópico controverso. Existe uma tendência entre esses indivíduos de apresentar perda de peso e controle de comorbidades significativos, porém menores que os observados na população obesa geral. A gravidade do déficit intelectual pode ser considerada no processo de decisão sobre a técnica cirúrgica mais adequada. A cirurgia bariátrica é factível e segura nesse grupo de indivíduos. Porém, mais estudos são necessários para aprofundar estas observações.

## INTRODUCTION

Bariatric surgery has become the standard treatment option for refractory morbid obesity. The observed overall impact of this surgery on obese patients has been found to be 40% regarding long-term reduction in mortality, 56% for coronary heart disease, 92% for diabetes complications and 60% for any type of cancer.^1^^,^^2^

Obesity and its related comorbidities are common among individuals with cognitive impairment, but the outcomes from bariatric surgery in this singular group remain uncertain.^3^^,^^4^^,^^5^^,^^6^^,^^7^ The majority of bariatric programs exclude patients with intellectual and/or developmental disabilities, from surgical indication. Only 6.2% of programs have not considered severe levels of impairment, i.e. intelligence quotient (IQ) of 50-70, to be a contraindication.^8^ Moreover, current guidelines emphasize the importance of a clear understanding among patients regarding the risks, benefits, outcomes and alternatives to surgery. This ability to give consent is possibly compromised in cognitively impaired individuals.^6^

## OBJECTIVE

To report the cases of two individuals with severe non-acquired cognitive impairment who underwent bariatric surgery.

## CASE REPORT

### Case 1

EGR, a 25-year-old male with Prader-Willi syndrome, whose IQ was 54, presented with a body mass index (BMI) of 55 kg/m^2^, associated with impaired glucose tolerance and walking disability. He underwent the Scopinaro procedure for biliopancreatic diversion, with uneventful postoperative evolution. The main caregiver during the postoperative phase was his mother, who chose to perform caregiving functions full-time.

One year after the surgery, he presented BMI of 38.5 kg/m^2^, i.e. a 55% loss of excess weight. His impaired glucose tolerance had been resolved; his ability to walk had developed; and he did not present any features of malnutrition. No cognitive evaluation test was performed after the surgery. Two years after the surgery, the family chose to leave the patient at a part-time non-profit institution for intellectually disabled people.

### Case 2

JLC, a 28-year-old male with Down syndrome, with IQ of 68, presented with a BMI of 41.5 kg/m^2^, associated with hypertension. He underwent Roux-en-Y gastric bypass, with uneventful postoperative evolution. The main caregivers during the postoperative phase were his mother and sister, who both chose to perform caregiving functions full-time.

One year after the surgery, he presented BMI of 26.7 kg/m^2^, i.e. a 90% loss of excess weight, with resolution of his hypertension. No cognitive evaluation test was performed after the surgery. One year after the surgery, the patient began to work part-time at a grocery store.

Table 1 summarizes the main clinical and laboratory outcomes relating to these two individuals.

**Table 1:**
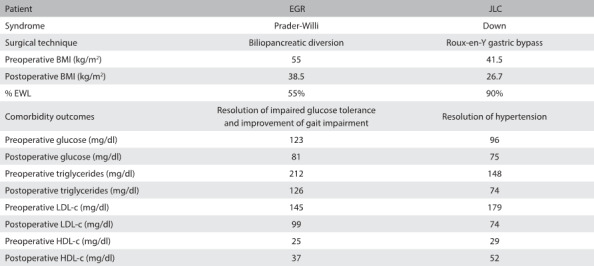
Outcomes from bariatric surgical procedures on two individuals with severe cognitive impairment

BMI = body mass index; % EWL = percentage of excess weight loss; LDL-c = low-density lipoprotein cholesterol; HDL-c = high-density lipoprotein cholesterol.

## DISCUSSION

Bariatric surgery among individuals with severe intellectual impairment remains a controversial topic. There is a tendency among these individuals to present significant weight loss and comorbidity control, but less than what is observed among individuals without cognitive impairment.^9^

A review of the literature on this subject was conducted through an online search for the Medical Subject Headings (MeSH) terms Prader-Willi syndrome, Down syndrome, intellectual disability and bariatric surgery, in MEDLINE (via PubMed) and LILACS (via BVS) (Table 2). After extensive online research, we identified six case series, five case reports, one matched-cohort study and one scoping review that evaluated bariatric surgery among individuals with severe cognitive impairment. Table 3 ^6^^,^^7^^,^^9^^,^^10^^,^^11^^,^^12^^,^^13^^,^^14^^,^^15^^,^^16^^,^^17^^,^^18^^,^^19^ summarizes the main articles found and their respective characteristics and levels of evidence, according to the Oxford classification.

**Table 2:**
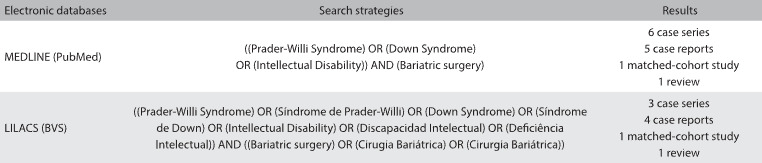
Database search results for bariatric surgery among individuals with severe cognitive impairment, conducted on November 14, 2016

**Table 3:**
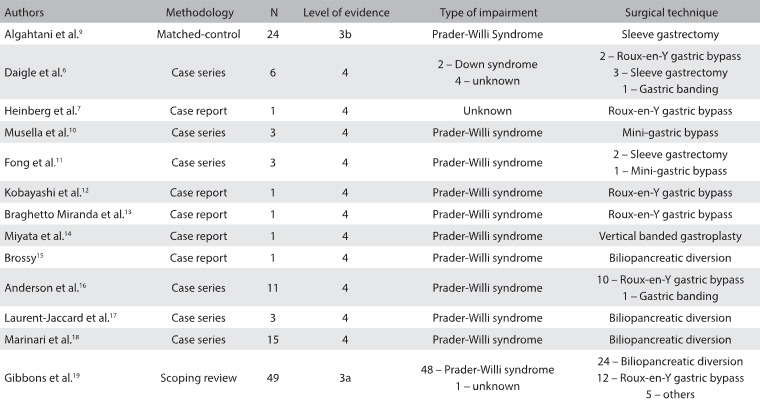
Main studies on bariatric surgery among individuals with severe cognitive impairment

N = number of individuals.

Interestingly, despite the much higher frequency of Down syndrome in the general population (one per 700 to 1,000 newborns), we observed that the vast majority of the studies included individuals with Prader-Willi syndrome, which occurs less commonly (1 per 10,000 to 25,000 newborns).^20^ In fact, only the study by Daigle et al.^7^ included two individuals with Down syndrome. Whether this finding is due to non-treatment of these individuals or underreporting of the treated cases remains to be determined.

There is a strong necessity for social support within this group of individuals, especially regarding family support and caregivers. Since these individuals do not present the capacity to understand and formally consent to such procedures, the main caregivers need to be directly asked about this topic. Social support must be emphasized postoperatively, to avoid loss of adherence to the long-term follow-up. ^6^^,^^7^^,^^8^

Since there is no consensus regarding which procedure is most appropriate, the severity of intellectual impairment may be taken into consideration in the decision-making process regarding which technique to use. Historically, predominantly restrictive procedures such as sleeve gastrectomy, Roux-en-Y gastric bypass (RYGB) and gastric banding were avoided among individuals whose intellectual deficit was more severe, such as in cases of Prader-Willi syndrome.^16^^,^^17^^,^^18^ However, this trend diminished over the years, to the point that nowadays Roux-en-Y and mini-gastric bypasses, and even sleeve gastrectomy, in which weight loss relies exclusively on a restrictive mechanism, are considered valid options. ^8^^,^^9^^,^^10^^,^^11^^,^^12^^,^^13^^,^^14^^,^^15^^,^^19^ There is newer evidence showing that restrictive techniques may be safe and effective in this group of subjects.^6^^,^^9^^,^^11^ In individuals whose deficit is slight or even borderline, predominantly restrictive procedures do not present any formal contraindications.^6^^,^^7^^,^^20^^,^^21^^,^^22^ With the sole exception of the study by Miyata et al.,^14^ in which an individual with Prader-Willi syndrome presented an initial improvement of metabolic and weight conditions, followed by progressive worsening, the vast majority of the studies have consistently observed significant improvements following a variety of techniques, regarding both metabolic features and weight loss, albeit to a lesser extent than what is observed in the general population with obesity when bariatric surgery is implemented.^6^^,^^7^^,^^8^^,^^9^^,^^10^^,^^11^^,^^12^^,^^13^^,^^15^^,^^16^^,^^17^^,^^18^^,^^19^

More studies are necessary, in order to provide evidence of higher quality that could lead to possible algorithms for this heterogeneous group of individuals. There is recent evidence that even mild cognitive impairment may play a role in the outcomes from bariatric surgery, such that it may lead to worse results and, especially, poor adherence to long-term follow-up.^23^^,^^24^ Nonetheless, a recent study by Rochette et al.^25^ observed a significant decrease in the prevalence of mild cognitive impairment after bariatric surgery. Whether this may be applicable to individuals with severe cognitive disability remains to be further investigated.

## CONCLUSION

Bariatric surgery is feasible and safe among cognitively impaired individuals, but further research is necessary.
